# “A constant struggle to receive mental health care”: health care professionals’ acquired experience of barriers to mental health care services in Rwanda

**DOI:** 10.1186/s12888-015-0699-z

**Published:** 2015-12-16

**Authors:** Lawrence Rugema, Gunilla Krantz, Ingrid Mogren, Joseph Ntaganira, Margareta Persson

**Affiliations:** Department of Community Health, School of Public Health, College of Medicine and Heallth Sciences, University of Rwanda, Kigali, Rwanda; Department of Public Health and Community medicine, The Sahlgrenska Academy, Gothenburg University, Gothenburg, Sweden; Department of Clinical Sciences, Obstetrics and Gynecology, Umeå University, Umeå, Sweden; Department of Biostatistics and Epidemiology, School of Public Health, College of Medicine and Health Sciences, University of Rwanda, Kigali, Rwanda; Department of Nursing, Umeå University, Umeå, Sweden

**Keywords:** Health seeking behavior, Mental disorders, Barriers and facilitators to care, Qualitative research, Content analysis, Rwanda

## Abstract

**Background:**

In Rwanda, many people are still mentally affected by the consequences of the genocide and yet mental health care facilities are scarce. While available literature explains the prevalence and consequences of mental disorders, there is lack of knowledge from low-income countries on health care seeking behavior due to common mental disorders. Therefore, this study sought to explore health care professionals’ acquired experiences of barriers and facilitators that people with common mental disorders face when seeking mental health care services in Rwanda.

**Methods:**

A qualitative approach was applied and data was collected from six focus group discussions (FGDs) conducted in October 2012, including a total of 43 health care professionals, men and women in different health professions. The FGDs were performed at health facilities at different care levels. Data was analyzed using manifest and latent content analysis.

**Results:**

The emerging theme *“A constant struggle to receive mental health care for mental disorders”* embraced a number of barriers and few facilitators at individual, family, community and structural levels that people faced when seeking mental health care services. Identified barriers people needed to overcome were: *Poverty and lack of family support, Fear of stigmatization, Poor community awareness of mental disorders, Societal beliefs in traditional healers and prayers, Scarce resources in mental health care* and *Gender imbalance in care seeking behavior*. The few facilitators to receive mental health care were: *Collaboration between authorities and organizations in mental health* and having a *Family with awareness of mental disorders and health insurance.*

**Conclusion:**

From a public health perspective, this study revealed important findings of the numerous barriers and the few facilitating factors available to people seeking health for mental disorders. Having a supportive family with awareness of mental disorders who also were equipped with a health insurance was perceived as vital for successful treatment. This study highlights the need of improving availability, accessibility, acceptability and quality of mental health care at all levels in order to improve mental health care among Rwandans affected by mental disorders.

## Background

Mental disorders are important causes of morbidity and disability in high and low-income countries [1]. Common mental disorders contribute to 14 % of the global burden of disease; however these disorders attract little attention in terms attention of health budgets allocated and staff trained specifically in mental disorders. Further are the associations with other health conditions such as communicable and non-communicable diseases, intended as well as un intended injuries underestimated [2]. A study by the World Health Organization (WHO) on mental health expenditure as percentage of the health budget in 89 countries shows that 79 % of the African countries included have no specific budget for mental health. Moreover, the African countries with a specific budget for mental health problems use less than 1 % of their total health budget on mental illnesses [3].

In low and middle income countries (LMIC), poverty and economic exclusion are associated with mental illness in the population [4]. About 75 % of people with mental disorders in low-income countries have insufficient or no access to evidence-based treatment [5]. The services are often located in cities and primary care workers are overburdened, and have insufficient skills or experience in mental health care [6]. Additionally, people with mental illness often face discrimination, are inappropriately incarcerated, and denied economic, social and human rights [7, 8]. The local language used to conceptualize mental illness may be dismissive and contribute to the stigma of mentally ill patients. Hence, people may be reluctant to seek health care as both self-confidence and self-esteem are reduced as a result of discrimination [9].

Rwanda, a low-income country in Central Africa, is deeply affected by the genocide of 1994 where an estimated 800,000 people were killed [10, 11]. It is known that conflict and war related traumatic episodes are associated with mental disorders [12]. The mental health policy in Rwanda is committed to promoting mental health awareness, providing mental health care and accessibility, and ensuring human resources for mental health; i.e. the Ministry of Health strives to reduce stigma and ensure community based services [13]. Yet, it remains a challenge to provide appropriate care for mental illness in Rwanda. In 2007, there was a remarkable increase in health service utilization and health access for the poor, with reduced payments from out-of-pocket [14], but the introduction of higher premiums in 2010 to get more finances to support the insurance program [15], increased difficulties for people to pay the annual premium for *Mutuelle de Santé* (health insurance scheme) for the nuclear family. Hence, the family members could not have access to health care during the discontinued period until payment of the insurance is made [16].

By 2011, there were a shortage of psychiatrists (0.05/100.000 inhabitants), psychologists (0.07/100,000 inhabitants) and few nurses (1.30/100,000 inhabitants) [17] available, the total number of mental health nurses were 293 in 2012 [18] and only two mental hospitals specialized in mental health care in Rwanda [13]. However, there are a few other mental clinics supported by the Congregation of the Brothers of Charity, providing mental health care and training mental health professionals [19], but taken together, the possibility of being offered evidence based treatment for common mental disorders is limited.

While available literature explains the prevalence and consequences of mental disorders, there is lack of knowledge on what barriers to evidence based care people suffering from mental disorders face. Therefore, this study sought to explore health care professionals’ acquired knowledge and experience of barriers and facilitators that people with mental disorder face when they are seeking mental health care services in Rwanda.

## Methods

### Study design

A qualitative study design, using focus group discussions (FGDs) with health care professionals, was employed. By using FGDs for data collection, the acquired experiences and opinions of the professionals could be disclosed. Thereafter, manifest and latent content analysis were applied for analyzing the collected data. The study was approved by Rwandan National Ethics Committee (FWA Assurance No. 00001973, IRB 00001497 of IORG0001100).

### Settings and participants

Three district hospitals and one mental hospital situated in the Southern part of Rwanda, one psychosocial center within the capital city Kigali and one mental hospital located on the outskirts of the capital city Kigali, were purposively selected to represent a variety of health care facilities providing health care to people with differing severity of mental disorders. Inclusion criteria were being a health care professional providing care to people with mental problems, regardless of participants’ sex, age, profession or length of work experience to cover a range of different experiences. All participants were Rwandans. The characteristics of the participants for each FGD are presented in Table 1.

**Table 1:** Characteristics of the study participants

FGD	Setting	Number of participants and sex distribution	Age	Number of participants with specialized training in mental health	Profession
			Mean (Min-Max)		
		(Male:Females)			
1	Mental hospital	10 (7:3)	36.6 (29–50)	10	Psychiatrist (1)
					Mental health nurses (8)
					Clinical psychologist (1)
2	District hospital	7 (1:6)	32.4 (26–38)	2	Mental health nurse (1)
					Clinical psychologist (1)
					General nursing (5)
3	Mental health center	7 (4:3)	32.0 (27–46)	7	Psychiatrist (1)
					Mental health nurse (4)
					Clinical psychologist (2)
4	District hospital	7 (4:3)	38.1 (28–59)	1	General nurses (5)
					Mental health nurse (1)
					General nurse for gender based violence (1)
5	Psychosocial center	6 (3:3)	32.5 (26–40)	6	Mental health nurses (5)
					Clinical psychologists(1)
6	District hospital	6 (1:5)	32.0 (27–44)	2	Mental health nurse (1)
					General nurses (4)
					Clinical psychologist (1)
Total		43 (20:23)	34.2 (26–59)	28	

*FGD* focus group discussion

### Interview guide

An interview guide was developed by the research team based on the study objectives and relevant literature. Examples of questions addressing the aim of this study were; *“From your professional experience, what do men and women do when they realize they suffer from mental disorders?”* and *“What barriers have you encountered among men and women as a reason for not seeking health care? In your opinion, what factors facilitate health care seeking for people with mental conditions?* Prior to the data collection, a pilot testing exercise was conducted to ensure that the questions in the interview guide were comprehensible. This resulted in minor corrections in the wording of a few questions.

### Data collection procedure

The head of each selected facility was informed about the study and asked to enable the recruitment of professionals for the FGD by informing staff of the study. An undisturbed space to conduct the FGD was arranged for at each setting. Before the start of each FGD, the research team presented the purpose of the study, informed the eligible participants of confidentiality and voluntary participation. All eligible individuals chose to participate and signed a consent form. Some background data of the participants were collected from responses to a short questionnaire.

The FGDs were performed during two weeks in October, 2012. All interviews were performed by a moderator and a co-moderator taking turns in being responsible for the interviews. A note-taker and an observer were also present during all FGDs. The language used in all FGDs was the Rwandan mother tongue, Kinyarwanda, and the interviews lasted on average 90 min. All FGDs were digitally recorded with participants’ permission. The number of FGDs was not predetermined. After six FGDs, data was considered saturated as no major new information had been revealed during the last FGD. All recordings were transcribed verbatim before translation into English for the purpose of analysis. Parts of the translated transcripts were re-translated back to Kinyarwanda to secure the accuracy of the translations.

### Analysis

By applying qualitative content analysis, differences and similarities were highlighted in the text and these were organised into codes, sub-categories, categories and theme. The theme is the latent content, mirroring the underlying meaning which cuts across all data [20].

First, the text was read through several times to capture a sense of the whole and identify content areas of the transcriptions. Thereafter, words, sentences and paragraphs (meaning units) related to each other by content and aim of the study, were identified. These meaning units were condensed and labelled with codes. All codes were then compared for similarities and differences, resulting in eight sub-categories and two categories. During the analysis, a theme emerged, i.e. the underlying thread illustrating the latent meaning of health care professionals’ acquired experiences of barriers and facilitators that people with mental disorder face when seeking mental health care services. The first and last author collaborated throughout the whole analysis. All researchers were involved in finalizing the analysis to ensure the objectivity of the findings.

## Results

An overview of the findings is presented in Table 2. The emerging theme “A constant struggle to receive health care for mental disorders” reflected a number of barriers and only a few facilitating factors. The following sub-categories mirror the barriers identified: “Poverty and lack of family support”, “Fear of stigmatization”, “Poor community awareness of mental disorders”, “Societal beliefs in traditional healers and prayers”, “Scarce resources in mental health care” and “Gender imbalance in care seeking behavior”. The few factors facilitating health care seeking were: “Collaboration between authorities and organizations in mental health” and “Family with awareness of mental disorder and health insurance” to receive treatment and follow-up.

**Table 2:** An overview of the theme, categories and their sub-categories

*Theme*	*Categories*	*Sub-categories*
*A constant struggle to receive health care for mental disorders*	*People facing numerous barriers when seeking health care for mental disorders*	*Poverty and lack of family support*
		*Fear of stigmatization*
		*Poor community awareness of mental disorders*
		*Societal beliefs in traditional healers and prayers*
		*Scarce resources in mental health care*
		*Gender imbalance in care seeking behavior*
	*The use of scarce facilitators to enable health seeking for mental disorders*	*Collaboration between authorities and organizations in mental health*
		*Family with awareness of mental disorders and health insurance*

The findings will be presented with an initial summary of the category, followed by a presentation of its included sub-categories. Findings are illustrated with quotations from the participants in the FGDs, presented in italics.

### “People facing numerous barriers when seeking health care for mental disorders”

The six sub-categories forming the category were composed of individual, societal, gender, cultural and structural barriers to get the needed mental care. According to experiences of the participants each of these barriers contributed to delay to get mental health care and treatments.

### Poverty and lack of family support

Participants, at all levels of care, perceived that poverty in general and fragmentation of families constituted a barrier to health care seeking for mental disorders. Poverty implied lack of necessary resources, such as transport and affording the annual health insurance premium. Some families had many members, so that coverage for health insurance for all family members was a struggle. Additionally, as a consequence of the genocide, a considerable number of families were fragmented; i.e. family members had been killed or were in jail, leaving behind no family member to support people with mental disorders. Another issue discussed was the troublesome situation of some teenagers who were not accepted by the family and unsure of which family they belonged to. Such cases were said to be among children born outside of wedlock or by parents of different ethnicity. Hence, they keep moving between families and consequently receive insufficient support from either family in case of them suffering from a mental problem.

The necessary treatment follow-up was perceived as less successful due to lack of family support and resources as the patient was not taken back to the health facility for needed follow-ups. Several examples were given of how necessary, but unaffordable medicines were prescribed and follow-up visits were not attended.

Furthermore, the professionals experienced that an individual with a mental problem might be regarded as someone not contributing to the daily life of the family, and merely causing increased burden. Consequently, the individual could be rejected from the family, which led to further deterioration of the mental status. Therefore, respondents expressed that individuals with no family support often wandered the streets in a worsened mental state, and with even smaller chances of receiving health care.

*“An ill person comes to hospital and is treated and gets better and returns back to hell. Because after we have treated them, we send them back to their families who have rejected them because of poverty. […] Even when they start getting better, these problems [mental problems] can re-occur, especially among people who have no families.”* (Female, District Hospital)

### Fear of stigmatization

According to the participants, having a mental disorder was stigmatizing, hence the fear of stigma caused delays in receiving treatment. The community was perceived as observant of any strange behavior in people and being labeled as a “mad man or woman” was feared.

Further, having a family member with a mental disorder affected the status of the entire family. The participants described cases where persons with mental problems were hidden from society by their family and later found in terrible conditions. Examples were also given of persons refusing referral to mental health facility for further assessment and treatment because they feared that their mental condition would be known to the community. It was mentioned that the mental hospital was strongly associated with severe mental illness. A participant suggested renaming the hospital to change the stigmatizing attitude locals associate with mental hospital, although not all agreed on this suggestion. Additionally, the participants expressed that individuals with mental problems, even if recovered, often seemed to have problems in their relationships; i.e. difficulties in finding a partner for marriage or if married, a divorce was often seen as a result of the mental condition.

The stigma related to mental disorders was well-known among health care professionals. Hence, the mental status of a patient could at times purposively be minimized by the professionals to ease the consequences of stigma for the individual.

*“It’s difficult to explain to a person that you have mental problems as mental illness is regarded as a heavy burden to carry in society [.…] A health care professional may lessen the severity of the mental condition as they are aware of the stigma attached to mental illness.”* (Female, Mental Hospital)

###  Poor community awareness of mental disorders

Poor awareness of mental disorders within the community was another factor perceived to delay health care seeking. The lack of trained staff in rural areas and low community awareness obstructed access to the needed care and contributed to delays. Examples were told of patients that could seek care in health centers for years before getting the appropriate diagnosis or referral for necessary treatment.

The professionals discussed and made comparisons with the available community-based educational programs successfully improving knowledge of gender-based violence, malaria and HIV. No such resources were available for improving community knowledge on mental health disorders, i.e. a situation fostering stigma and contributing to delays in mental health care seeking.

“*I always ask people how long it took them to come to us. You find that it took a long time like two or three years, taking Paracetamol prescribed by a health center. They [health care staff at health centers] do not have enough knowledge on when to process the transfer. This is a big challenge, indeed.”* (Male, Mental Hospital)

### Societal beliefs in traditional healers and prayers

Seeking help from traditional healers was discussed as a common phenomenon among people with mental disorders, hence delaying heath care seeking. Exorcism to cure individuals was repeatedly mentioned as a result of community beliefs in evil spirits. Some participants also cited that occasionally patients engaged in prolonged prayers in the nearby church to seek recovery by “the power of prayers”, also resulting in delayed counselling. The general notion among the professionals was that the community had exaggerated expectations of the power of healers and prayers. Consequently, the mental health care was regarded as the last resort when no other actions taken had caused improvement.

*“Some people still have the mentality that even though they went to hospital and got treatment, they continue being treated by the nearest traditional healer or they may go to prayer meetings.”* (Male, District Hospital)

### Scarce resources in mental health care

Participants expressed that the prevalence of mental disorders was a larger problem than recognized by the decision makers and they experienced that the number of persons seeking care was constantly increasing. The high mortality rates due to suicide and homicide in the community were also perceived to be related to mental disorders.

Most professionals were of the opinion that mental health services were poorly funded, had a poor infrastructure; few specialized facilities were available and these most often lacked qualified professionals. Consequently, the perceived needs and the available resources did not match which also contributed to delays receiving health care.

“*So far there is one neurological hospital in Rwanda. District hospitals do not hospitalize patients but transfer them to a referral unit. There is no mental health staff available to deal with cases at the grassroots level. All of those challenges contribute to increasing the cases of mental illness instead of decreasing them.”* (Male, Mental Hospital)

### Gender imbalance in care seeking behavior

The general notion among professionals, were that more males avoided seeking help for mental disorders and tended to minimize their mental problem. Instead of seeking health care, males tried other solutions to ease or escape their problems, such as frequent use of drugs and alcohol.

*“For a man, a request for help means acceptance of failure to solve his problem. This means before accepting failure [he] tries many possible ways to solve his problem. This does not mean men are not ill, but rather they resort to alcoholism and they are always reluctant to come for treatment.”* (Female, Mental Hospital)

On the other hand, participants described that more women were seeking treatment for mental problems. However, they found it difficult to actually know whether women were more prone to seek mental health care or if it was due to other reasons. Women were perceived as being more willing to adhere to appointments and treatment than men.

“*According to the statistics established after every commemoration period and the number of people with trauma that we receive every year, it is clear that females represent the larger number of affected persons.”* (Female, Mental Hospital)

### “The use of scarce facilitators to enable people’s health seeking for mental disorders”

Health care professionals experienced only some facilitators that enabled people to receive mental health care. These facilitators also addressed different levels within the community.

### Collaboration between authorities and organizations in mental health

Some participants expressed that community health workers provided information concerning mental health to the community, although no official awareness raising program on mental health existed. Also, charity organizations supported some mental health care centers and in this way contributed to provide available and relevant care. The police often brought individuals wandering in the street with suspected mental disorders to a health facility to be assessed and treated. Other collaborators experienced in improving the situation of mental health in the community were the trauma counselors. These are specialized groups helping traumatized people, who live in the community. Some participants appreciated the role of the church, while others did not. Some expressed that churches could be helpful by providing hope through faith based psychotherapy*,* while others instead thought that the church should recommend patients with mental disorders to seek for medical treatment.

“*In prayers, there is what could be called spiritual psychotherapy and even experts recognize that fact and medical caregivers use psychotherapy which is faith-based. If I say that such faith-based psychotherapy is not helpful, some people would get upset.”* (Male, Mental Hospital).

### Family with awareness of mental disorders and health insurance

Most participants stressed that a triplet of closely related factors were vital to receive mental health care for mental disorders. These factors were having a supportive family who were aware of mental disorders and provided health insurance for all family members. This meant that health insurance covered the required treatment; the individual had somewhere to live and received support for treatment and follow-ups. Having insurance only, but no understanding or support from the family was not perceived as helpful. Having a supportive family, but without insurance was perceived to be just as bad since the individual would not receive the necessary treatment.

*“Once at the hospital, a patient can be treated with the help of health insurance […], however knowing that the patient has no family to provide food to eat or will have insufficient meals is a problem. A patient can come for a first visit, but miss the follow-up visit if abandoned by the family.”* (Male, Mental Hospital)

## Discussion

This is the first study in a Rwandan setting addressing health care professionals’ acquired experiences of the barriers and facilitators that people with mental disorders face when they are seeking mental health care services in Rwanda.

The emerging theme “*A constant struggle to receive health care for mental disorders*” reflected health care professionals’ acquired experience of barriers and facilitators. The barriers people with mental disorders faced when seeking care covered several levels within society. At the individual level, people feared the stigma associated with mental disorders. Further, there were several barriers at the family level, such as poverty that implied lack of family resources which negatively affected the possibility to receive mental health care. Further factors to overcome were the poor awareness of mental disorders at community level and the community’s strong beliefs in healers and prayers, which contributed to further delays in help seeking. Also, gender imbalances in health seeking behavior was noticed at community level while gender roles are constructed on the structural level and hereby influence all levels of societal organization. Additionally, barriers such as underfinanced mental health services and lack of trained staff were emphasized, as structural level factors. The few identified facilitators also addressed various levels within the society. The family level was emphasized for its importance for successful treatments, but also the benefits of collaborating at different levels within the society were stressed.

The findings of this study will be discussed in light of the “*The Right to Health”* concept*,* which includes Availability, Accessibility, Acceptability and Quality of care (AAAQ) [21]. Availability implies that health facilities, essential medicine, supplies and trained health care professionals should be available in enough quantity [21]. Accessibility means that health services should be accessed with no discrimination also to the most vulnerable groups. Health care should be economically and geographically accessible and in line with people’s needs [21]. Acceptability indicates that health services have to respect medical ethics, indigenous culture and gender sensitivity of health service users [21]. Quality of care concerns providing health services by qualified staff who ensures safe and relevant treatment [21]. The health professionals in this study expressed that structural barriers such as poor resources, lack of educational interventions and shortage of trained staff contributed to delay and exacerbated peoples’ access to mental health care. Interventions targeting other specific areas have been successful in Rwanda, for example, after the Government intervention in malaria treatment, which provided insecticide treated bed nets to people and improved access to medications, a more than 50 % decrease of malaria cases was reported [22]. Similarly, awareness campaigns committing resources to mental health care could mean successful interventions contributing to better accessibility, availability and quality improvements to the help seeking situation experienced by the participants in this study.

Geographical accessibility and affordability were important barriers raised in our study. In Rwanda the health insurance (*Mutuelle de Santé)* ensures financial protection of individuals when accessing health care without being impoverished by out of pocket spending [14]. Despite efforts made to achieve a high coverage of Mutuelle de Santé in the population, the poorest part still show a lower utilization of health care than those better off [23]. According to the WHO, poverty and mental disorders relate negatively as those with mental disorders have limited employment opportunities, which will increase deprivation and delays health care seeking due to lack of financial means [24, 25]. On the other hand, the association between mental disorders and poverty goes both ways i.e. there is a risk of mental disorders due to poverty as well [4].

The acceptability within the community of available mental health care is aggravated by the stigma attached to mental disorders. After the Rwandan genocide, several studies show that a considerable number of citizens of different ages suffer from mental disorders related to traumatic episodes [11, 26–30]. Yet, the stigma attached to mental illness prevents health seeking [31, 32], retarding early detection despite the widely recognized benefit of early intervention [33].

In addition, individuals often seek relief for their health problems from traditional healers [34]. Improving the acceptability of mental care requires better community awareness of mental health problems and a change of the widespread beliefs in traditional healers. In other African countries such as Kenya, the use of traditional healers is prominent and referral of patients to hospital only happen if they show no improvement after the healer’s treatment [35]. In Uganda, over 80 % of patients with psychosis use both medical services and traditional healers [36]. No studies that address the Rwandan use of healers in mental disorders are available, however there is a Rwandan study showing that 37 % of women with breast cancer consult traditional healers [34].

Lack of qualified staff may endanger the quality of care [21]. In Rwanda, there are few qualified and trained staff in mental health services [17], which negatively impact the quality of services provided and outcome of treatments. A study conducted among genocide survivors show that individuals prefer to seek mental care service at the district hospital instead of their nearby health centers as they perceive quality to be poor at the health centers [37].

More women than men were perceived to seek help for mental disorders according to our participants. It is described elsewhere that more women than men suffer from mental disorders [38, 39]. A Rwandan study five years after the genocide shows that more women than men report current depression [40]. However, contrary findings are shown. A study 14 years after the genocide report that men present higher prevalence of posttraumatic stress disorders (PTSD) compared to women [11]. These contradictory findings highlight the problem of estimating the prevalence of mental disorders in Rwanda. Accordingly, more research is needed to provide a comprehensive status of the situation of mental disorders to enable the ministerial level to improve resources at the health care facilities’ level to match the needs of the people.

The usefulness of a supportive family and awareness of mental disorder have been described in other studies as facilitators for seeking mental health treatment [41, 42]. The combination of these factors was stressed as vital for a successful treatment in our study. In addition, it is shown that when the individual is aware of his/her problem, it becomes easier to recognize and accept care and sources of social support [43]. While prioritization in low resourced settings can depend on competing needs, the ongoing long standing ignorance and low priority given to mental health care services may be regarded as withholding needed resources to the people, i.e. could be regarded as a violation of human rights. Having access to medical care is emphasized in the *“The Right to Health*” concept as a basic human right [44, 45].

The experiences derived from the previous interventions successfully reducing malaria [22], could be used to perform similar interventions addressing mental disorders. Such an intervention could decrease the societal stigma attached to mental disorder among other barriers and consequently reduce the barriers to mental health seeking.

### Methodological considerations

One strength of our study is the purposive selection of participants working at different levels of the health care system, and also including different medical professions and both sexes as well as rural and urban areas of the country.

Trustworthiness in qualitative research means addressing credibility, dependability, transferability and confirmability of the findings [46, 47]. Researchers with expertise in public health and qualitative research, of different professions and cultural backgrounds have collaborated throughout the whole project which contributes to secure the trustworthiness of the findings. To ensure confirmability, two of the authors with different cultural background and professional experience (LR and MP) analyzed all translated data and discussed the findings throughout the whole analysis.

During the FGDs, efforts were made to ensure that all participants felt they could contribute with their professionally acquired experiences; this to address power asymmetry between interviewers and interviewees which can affect findings [48]. Translations from Kinyarwanda to English were done by a professional translator not part of the research team, and parts of the transcripts have been re-translated to make sure that the translation was accurate and that no important data had been lost. Also, a thorough description of the procedures is provided to improve the trustworthiness of the findings. By addressing the described aspects of trustworthiness, and our knowledge of the health care services organisation and practice, we trust that our findings do reflect experiences of other health care professionals meeting individuals with mental disorders at different levels of the health care organisation in Rwanda.

## Conclusions

From a public health perspective, this study revealed important findings of the numerous barriers and the few facilitating factors available to people seeking health for mental disorders. Having a supportive family with awareness of mental disorders who also were equipped with a health insurance was perceived as vital for successful treatment. This study highlights the need of improving availability, accessibility, acceptability and quality of mental health care at all levels in order to improve mental health care among Rwandans affected by mental disorders.
